# The effect of peer relationship on subjective well-being among Chinese middle school students: a chain mediation model

**DOI:** 10.3389/fpsyg.2025.1495506

**Published:** 2025-09-26

**Authors:** Lijun Wang, Weiqin Xiang, Ziyi Yang, Mengfei Cheng, Jingyi Shi, Zhendong Wan

**Affiliations:** ^1^School of Educational Science, Anhui Normal University, Wuhu, China; ^2^School of Psychology, Nanjing Normal University, Nanjing, China

**Keywords:** subjective well-being, peer relationship, dispositional optimism, emotion regulation strategy, Chinese middle school students

## Abstract

**Objective:**

To explore how peer relationship relates to subjective well-being in Chinese adolescents, and to examine the mediating roles of dispositional optimism and two emotion-regulation strategies (cognitive reappraisal, expressive suppression).

**Methods:**

A total of 1897 middle school students aged 11–19 (14.86 ± 1.69, 51.3% males) from Anhui Province, China, completed four questionnaires, by using a cluster convenience sampling method. The Adolescent Peer Relationship Scale, Index of Well-being, Emotion Regulation Questionnaire, and the Life Orientation Test-Revised were used to assess peer relationship, subjective well-being, emotion regulation ability and dispositional optimism, respectively. Data were statistically analyzed using descriptive statistics, correlation analysis, group comparisons, and mediation analysis.

**Results:**

Peer relationship significantly and positively predicted the subjective well-being of middle school students (*β* = −0.339, *p* < 0.001). Dispositional optimism (*β* = −0.391, *p* < 0.001) and cognitive reappraisal (*β* = −0.161, *p* < 0.001) were both found to partially mediate this relationship. Specifically, dispositional optimism significantly predicted both cognitive reappraisal (*β* = 0.242, *p* < 0.001) and subjective well-being (*β* = 0.260, *p* < 0.001), and cognitive reappraisal also positively predicted subjective well-being (*β* = 0.191, *p* < 0.001). Mediation analysis revealed that both dispositional optimism and cognitive reappraisal significantly mediated the relationship between peer relationship and subjective well-being. The mediating effect included three distinct paths: (1) the independent mediating effect of dispositional optimism (effect = −0.489), (2) the independent mediating effect of cognitive reappraisal (effect = −0.149), and (3) the chain mediating effect of dispositional optimism and cognitive reappraisal (effect = −0.087).

**Conclusion:**

This study found that peer relationship is positively associated with subjective well-being of middle school students. Furthermore, this relationship is explained through two key mechanisms: the independent mediating roles of dispositional optimism and cognitive reappraisal, and the sequential mediation pathway involving both. These findings underscore the importance of fostering positive peer relationship and cultivating psychological strengths such as optimism and adaptive emotion regulation strategies to enhance adolescents’ well-being.

## 1 Introduction

Within the Chinese sociocultural context, even under different sociocultural contexts, the perception of well-being is all related to an individual’s physical and mental health development. Subjective well-being (SWB) refers to the global judgments of life satisfaction and emotional responses (Diener et al., 1999), and is closely linked to mental health (Yue et al., 2006). For middle school students, SWB serves as a critical indicator of physical and psychological development as well as academic and life quality (Li et al., 2012; Cadime et al., 2016). Higher levels of SWB are linked to stronger family relationships, better interpersonal functioning, and improved social adaptability (Chu et al., 2023; Cai and Hu, 2024). However, a study on the social and emotional abilities of Chinese adolescents showed that only 60% of 10-year-olds and 35% of 15-year-olds report predominantly positive experiences of well-being (Yuan et al., 2021). These data suggest a decline in adolescents’ perceived well-being with age. Therefore, it is imperative to investigate the factors associated with SWB of Chinese middle school students along with its underlying mechanisms.

According to the personality-situation interaction theory (Diener et al., 1999), an individual’s SWB is influenced by the interaction between environmental and personal factors. In the school microsystem, peer interactions constitute a critical situational influence for middle school students. Peer relationship is defined as the patterns and processes of interactions with peers encompassing both positive and negative aspects (Hay et al., 2004). Research has shown that middle school students with stronger peer relationship tend to receive greater peer support, face a lower risk of experiencing harm at school, and report higher levels of SWB (Zhang et al., 2019). As a key protective factor in adolescent development, positive peer relationship offer a sense of inclusion and belonging, and adolescents who experience higher-quality friendships and more harmonious peer environments tend to report greater levels of positive emotions, such as joy and relaxation, which in turn contribute to enhanced well-being (Griffith et al., 2019; Niu et al., 2021). Based on these, the present study proposes H1: peer relationship exert a direct effect on the SWB of middle school students.

Among individual internal factors, personality trait is one of the most reliable and effective predictors of SWB (Diener et al., 1999). Dispositional optimism, defined as the relatively stable, generalized expectation that positive outcomes will occur across important life domains (Scheier and Carver, 1987), can effectively maintain individual mental health (Fitzpatrick, 2017). Peer relationship affects adolescents’ optimism level (Orejudo et al., 2012), and studying in a positive social relationship atmosphere and being recognized and accepted by classmates will promote the formation and development of students’ optimistic personality (Oberle et al., 2018; Tetzner and Becker, 2019; Zou et al., 2022). According to Vroom’s expectation theory (Vroom, 1964), optimists tend to embrace more active lifestyles, more active coping strategies, and seek good social adaptation and personal development due to their heightened expectations for the future. Existing literature suggests that optimism can significantly positively predict adolescents’ life satisfaction, which is one of the components of SWB (Zou et al., 2022). It can be speculated that peer relationship may enhance SWB by facilitating the formation of dispositional optimism. Besides, adolescents’ ability to regulate emotions is a major factor in maintaining their physical health and SWB (Gross, 2015). Emotion-regulation strategies—cognitive reappraisal and expressive suppression—effectively reduce individual negative emotions and exert a lasting impact on enhancing SWB (Zhao et al., 2014; Yan et al., 2022; Zou et al., 2022). Cognitive reappraisal involves reframing one’s interpretation of an emotional event and generating more adaptive appraisals to alter its impact, while expressive suppression refers to the internal and external efforts to inhibit emotional responses once they arise (Gross, 1998). The emotion regulation process model holds that early strategies (such as cognitive reappraisal) are more effective than late strategies (such as expressive suppression). Previous studies have found that cognitive reappraisal strategies are positively correlated with positive emotions, life satisfaction and other positive outcomes, and can significantly predict individual’s SWB, while expression inhibition strategies are positively correlated with social anxiety, depression and other negative states (Chen and Wang, 2014; Chen et al., 2016; Zhang et al., 2022). Moreover, individuals who are better at using emotion regulation strategies tend to have higher SWB (Zhang et al., 2020). Based on these, we hypothesize H2: dispositional optimism and emotion regulation strategies independently mediate the relationship between peer relationship and SWB.

Dispositional optimism is correlated with adolescents’ peer relationship and SWB. Adolescents with a high degree of optimism tend to have better peer relationship and experience higher SWB (Zhang et al., 2022). Personality traits such as optimism influence how individuals regulate and respond to emotional experiences, thereby shaping adaptive development (Carver and Connor-Smith, 2010; Segerstrom and Smith, 2019), and individuals with high levels of extraversion and agreeableness show higher well-being (Anglim et al., 2020). Moreover, dispositional optimism has been found to correlate significantly with multiple dimensions of emotion regulation. Specifically, the optimism component of this trait is positively correlated with all six dimensions of emotion regulation strategies (Chen and Fu, 2013). According to Fredrickson’s broaden-and-build theory, positive emotions expand individuals’ cognitive and behavioral repertoires and help build lasting psychological resources. Optimists, through the use of cognitive reappraisal, are more likely to generate positive emotions, which can initiate an positive cycle of “positive emotion–resource accumulation–enhanced well-being” (Fredrickson, 2004). When facing negative situations of varying intensity, optimistic individuals are more inclined to adopt flexible and adaptive reappraisal strategies to manage emotional distress, whereas pessimists often exhibit rigid emotional coping patterns (Wang and Wang, 2023). Thus, students with higher levels of optimism are better at using cognitive reappraisal strategies to deal with external troubles and promote their SWB. Previous studies have also shown that cognitive reappraisal strategies partially mediate the relationship between personality traits and SWB (Kobylińska et al., 2020). As an important protective factor for adolescent adaptation, dispositional optimism will have a certain impact on emotional regulation, and the differences in emotional regulation can also reflect the differences in dispositional optimism of adolescents (Zou and Yuan, 2021). In conclusion, this study hypothesizes H3: dispositional optimism and emotion regulation strategies play a chain mediating role between peer relationship and SWB.

## 2 Methods

### 2.1 Participants

A total of 2,017 questionnaires were distributed to students across multiple middle schools in Anhui Province, China, using a cluster convenience sampling method. Inclusion criteria were as follows: (a) enrollment in grades 7–12 (ages 11–19) as full-time students; and (b) absence of diagnosed physical or mental health conditions that could impair comprehension or response validity. Participants with diagnosed psychiatric, developmental, or neurological conditions were excluded from the sample, based on school health records and teacher reports, to avoid potential confounding effects on emotional or interpersonal functioning. Exclusion criteria included: (a) incomplete demographic information (e.g., missing gender or grade); (b) partially completed questionnaires; and (c) inattentive or patterned responses (e.g., straight-lining, zigzag patterns, or inconsistent answers to reverse-scored items). After applying these criteria, 1897 valid questionnaires were retained, yielding a valid response rate of 94.10%. The final sample comprised 973 male and 924 female students. Participants were distributed across grade levels as follows: Grade 7 (*n* = 397), Grade 8 (*n* = 343), Grade 9 (*n* = 337), Grade 10 (*n* = 286), Grade 11 (*n* = 265), and Grade 12 (*n* = 269). Among them, 995 were only children and 902 were non-only children. The average age of participants was 14.86 years (SD = 1.69), ranging from 11 to 19 years.

Prior to data collection, informed consent was obtained from all participants, their parents and teachers. Participants were assured of the confidentiality and anonymity of their responses. All procedures were conducted in accordance with ethical guidelines for research involving human participants.

### 2.2 Measures

#### 2.2.1 Adolescent Peer Relationship Scale (APRS, Chinese version)

The Adolescent Peer Relationship Scale (APRS), developed by Guo and Zhang (2004), was used to assess adolescents’ self-perceived peer interactions, which is applicable to primary and secondary school students aged 7–18 years old. This scale consists of 22 items and evaluates four dimensions: social anxiety, social avoidance, peer acceptance, and conflict resolution, including statements such as “I pay attention to how other classmates see me,” “I do not like being at school.” Responses are rated on a 4-point Likert scale, with 7 items reverse-scored. Higher total scores indicate lower-quality peer relationship. The Cronbach’s alpha coefficient of this scale in this study was 0.75, indicating that the scale has good internal consistency.

#### 2.2.2 Index of Well-Being (IWB, Chinese version)

The Index of Well-being (IWB), developed by Campell and later translated and revised by Li and Zhao (2000), was used to assess participants’ SWB. The scale comprises two components: a general affective index (8 items) and a life satisfaction item (1 item). The first part uses 8 pairs of bipolar adjectives (e.g., “happy-painful,” “valuable-useless”) rated on a semantic differential scale. The second part consists of a single item measuring overall life satisfaction, such as “I am very dissatisfied with my life in general—I am very satisfied with my life in general,” rated on a 7-point Likert scale. The total score is calculated by averaging the affective index and the life satisfaction score equally (1:1), resulting in an overall index ranging from 2 to 14. Higher score indicates greater levels of current SWB for individuals. In this study, the IWB demonstrated good internal consistency, with a Cronbach’s alpha coefficient of 0.83.

#### 2.2.3 Emotion Regulation Questionnaire (ERQ, Chinese version)

The Emotion Regulation Questionnaire (ERQ), developed by Gross and translated and revised by Li et al. (2010), was employed to assess the utilization of two emotion regulation strategies (cognitive reappraisal and expressive suppression) in individuals’ daily lives. The ERQ consists of 10 items rated on a 7-point Likert scale, such as “I control my emotions by changing the way I think about the situation. Higher scores indicate more frequent use of the regulation strategy and greater emotion regulation ability. In this study, the ERQ demonstrated good internal consistency, with a Cronbach’s alpha coefficient of 0.82.

#### 2.2.4 The Life Orientation Test-Revised (LOT−R, Chinese version)

The Life Orientation Test-Revised (LOT-R), developed by Seheier and Carver and translated and revised by Wen (2012), was used to measure individuals’ levels of dispositional optimism. The scale consists of 6 items rated on a 5-point Likert scale, including 3 positive descriptors such as “I frequently anticipate favorable outcomes in uncertain circumstances” and 3 negative descriptors such as “I never expect things to go the way I want them to go.” Higher total scores indicate higher levels of dispositional optimism. In this study, the LOT-R demonstrated acceptable internal consistency, with a Cronbach’s alpha coefficient of 0.78.

### 2.3 Statistical analyses

The statistical analysis of the data was conducted using SPSS 26.0 and the PROCESS macro, with a data missing rate lower than 3.3% for all single variables. The mean interpolation method was employed to handle missing data. The specific steps of data analysis were as follows: (1) Harman’s single-factor test was performed to assess common method bias; (2) Descriptive statistics, correlation analyses, and group comparisons across demographic variables were conducted; (3) A chain mediation model was tested using Model 6 of the PROCESS macro and Bootstrap method by repeated sampling 5,000 times, with dispositional optimism and emotion regulation strategies as mediators between peer relationship and SWB.

## 3 Results

### 3.1 Common method variance test

A Harman one-way analysis of variance (Podsakoff et al., 2003) was conducted to test for the presence of common variance. The analysis identified 10 factors with eigenvalues greater than 1. However, the first factor accounted for 19.1% of the total variance, falling below the threshold of 40%. This finding suggests that there is no significant presence of common method bias in the study.

### 3.2 Descriptive statistics and correlations

Descriptive statistics and zero correlations for all study variables are presented in Table 1. As expected, peer relationship^1^ was positively correlated with SWB, dispositional optimism, and cognitive reappraisal. Conversely, peer relationship negatively correlated with expressive suppression. Dispositional optimism was positively correlated with SWB, both dimensions of emotion regulation strategies, and negatively correlated with expressive suppression. Additionally, SWB was positively correlated with cognitive reappraisal strategies and negatively correlated with expression expressive suppression.

**Table 1:** Descriptive statistics and correlations for the study variables.

Variable	PR	DO	CR	ES	SWB
PR	1				
DO	−0.386^**^	1			
CR	−0.257^**^	0.302^**^	1		
ES	0.105^**^	−0.198^**^	0.211^**^	1	
SWB	−0.491^**^	0.445^**^	0.358^**^	−0.090^**^	1
M	1.946	3.744	4.835	4.077	9.487
SD	0.467	0.629	1.037	1.281	2.254

*N* = 1897, *p < 0.05, **p < 0.01; peer relationship = PR, dispositional optimism = DO, cognitive reappraisal = CR, expressive suppression = ES, subjective well-being = SWB.

### 3.3 The chain mediation test

In this study, we estimated the mediating effect across 5,000 replications of samples, controlling for gender, age, and grade. Additionally, we tested the mediating effect with a 95% confidence interval. We further examined the chain mediating effects of dispositional optimism and cognitive reappraisal between peer relationship and SWB. As shown in Table 2, the results indicate that peer relationship can positively predict SWB (*β* = −0.339, *p* < 0.001), dispositional optimism (*β* = −0.391, *p* < 0.001) and cognitive reappraisal strategy (*β* = −0.161, *p* < 0.001). Moreover, dispositional optimism can positively predict cognitive reappraisal strategy (*β* = 0.242, *p* < 0.001) and SWB (*β* = 0.260, *p* < 0.001), while cognitive reappraisal strategy can positively predict SWB (*β* = 0.191, *p* < 0.001).

**Table 2:** Outcomes of chain mediation models.

variables	DO		CR		SWB	
	*β*	*t*	*β*	*t*	*β*	*t*
Gender	0.078	3.695^***^	−0.043	−1.995^*^	−0.043	−2.334^*^
PR	−0.391	−18.457^***^	−0.161	−6.863^***^	−0.339	−16.624^***^
DO			0.242	10.277^***^	0.260	12.557^***^
CR					0.191	9.702^***^
R	0.393		0.341		0.594	
R^2^	0.155		0.116		0.353	
F	173.454^***^		82.932^***^		257.912^***^	

Gender is dummy coded, with male = 0 and female = 1. *p < 0.05, ***p < 0.001.

**Figure 1:**
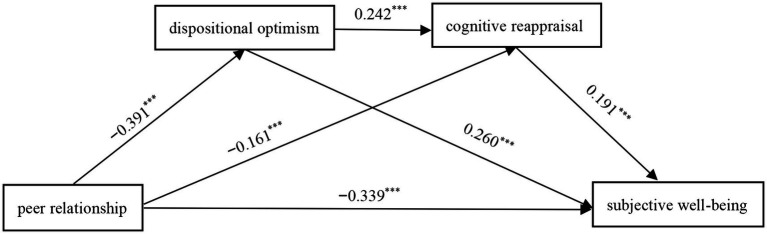
The effect of chain mediation model (standardized regression coefficients).

The mediating effect was tested using deviation-corrected non-parametric percentage Bootstrap. The results of the mediation analysis for dispositional optimism revealed that the 95% confidence interval for the mediation effect did not include zero (95%CI = [−0.590, −0.392]), indicating that dispositional optimism mediated the effect of peer relationship on SWB, with a mediation effect of −0.489, accounting for 20.73% of the total effect. The results of the mediation analysis for cognitive reappraisal revealed that the 95% confidence interval did not include zero (95%CI = [−0.210, −0.079]), indicating that cognitive reappraisal mediated the effect of peer relationship on SWB, with a mediation effect of −0.149, accounting for 6.32% of the total effect. The chain mediation analysis results for dispositional optimism and cognitive reappraisal revealed that the 95% confidence interval did not include zero (95%CI = [−0.119, −0.059]), indicating that the chain mediation effect was significant with a mediation effect of −0.087, accounting for 3.69% of the total effect (see Table 3; Figure 1).

**Table 3:** The direct, indirect, and total effect of chain mediation model.

Paths	Estimate	Ratio	95% confidence interval
PR − DO − SWB	−0.489	20.73%	[−0.590, −0.392]
PR − CR − SWB	−0.149	6.32%	[−0.210, −0.079]
PR − DO − CR − SWB	−0.087	3.69%	[−0.119, −0.059]
Total effect	−0.725	30.73%	[−0.884, −0.608]

## 4 Discussion

### 4.1 Peer relationship and subjective well-being

Our results confirmed Hypothesis 1, which suggests that peer relationship are strongly and positively associated with SWB. Specifically, better peer relationship correspond with higher levels of SWB, aligning with prior researches (Griffith et al., 2019; Chen et al., 2020; Niu et al., 2021). From an ecosystem theory perspective, peer relationship is part of the microsystem and holds significant adaptive value for individual development (Chai et al., 2018). Consequently, the more time students spend in school, the closer their relationships with peers tend to become, which in turn facilitates more positive emotional experiences and reduces the likelihood of negative events thereafter. Positive peer relationship offers adolescents enhanced recognition, support, and a sense of security (Brezicha and Miranda, 2022), which in turn foster positive emotions such as happiness and contentment. When students encounter fewer negative peer-related experiences and cultivate more supportive peer networks, their psychological adjustment and positive appraisals of life increase, ultimately improving individuals’ SWB.

### 4.2 The mediating role of dispositional optimism and emotion regulation strategy

This study partially supports H2 by demonstrating that both dispositional optimism and cognitive reappraisal independently mediate the positive association between peer relationship and SWB. This finding highlights dispositional optimism as a key positive personality factor influencing adolescents’ SWB, aligning with previous research asserting that “greater optimism begets greater happiness” (Serrano et al., 2020). The conjoint influence of peer relationship and dispositional optimism on SWB confirms the interdependence of external environmental factors and individual personality traits. During social interactions, students who enjoy high-quality peer relationship tend to cultivate elevated levels of dispositional optimism, which in turn enhances their SWB. This might be related to the positive attribution style of optimists (Yuan and Zhang, 2007). For example, optimists are good at making favorable attributions to important life events. They tend to attribute negative events to external, transient, and specific causes while attributing positive events to internal, stable, and global factors. Furthermore, students experiencing greater peer acceptance and more harmonious peer ties often adopt an optimistic outlook on life (Grandchampa et al., 2021; Zhang, 2017). Such a positive mindset and corresponding behaviors are conducive to an individual’s social interactions with others. They can unintentionally spread positive energy to the surrounding environment, thereby gaining more respect, understanding, and higher social support from others. This enables individuals to make full use of social support resources to better adapt to social life, improve personal quality of life, and thus have a higher level of life satisfaction and experience a stronger sense of SWB (Qi, 2013; Liu et al., 2022).

Specifically, higher-quality peer relationship predicted more frequent use of cognitive reappraisal (one of emotion regulation strategies), which in turn is linked to greater SWB. This finding is consistent with previous study (Hu et al., 2019). According to the compensatory theory of mind (Vîslă et al., 2016), individuals can use cognitive reappraisal strategies to compensate for deficiencies, buffer the adverse effects brought by stressful situations, develop individual psychological resilience, effectively improve living standards by actively constructing psychological resources, and ultimately enhance SWB. This enables them to tap into positive resources for coping with negative emotions and ultimately experience higher SWB (Chai et al., 2018; Chen and Wang, 2014; Wang and Jia, 2021). When students have high-quality peer relationship, they can receive more emotional support, use cognitive reappraisal strategies to reinterpret negative emotions, and effectively adjust their internal cognition. It is worth noting that expressive suppression did not significantly predict SWB, which may be related to the cultural adaptability of expressive suppression strategies. In traditional Chinese culture, suppressing the expression of one’s own emotions or behaviors is regarded as a good personal quality to cultivate and does not emphasize the autonomy of self-expression. “think twice before speak or act” (谨言慎行) is a typical representative of this phenomenon. Chinese people generally regard expressive suppression as a common behavior, and its negative effects are weakened, not enough to have a significant impact on daily life (Soto et al., 2011).

### 4.3 The chain mediating of dispositional optimism and emotion regulation strategy

Last but not least, this study has also found that dispositional optimism and cognitive reappraisal strategies have a chain mediating effect between peer relationship and SWB in Chinese middle school students, confirming Hypothesis 3. Positive peer relationship may foster optimistic personality traits of middle school students, which in turn promote the use of cognitive reappraisal strategy, ultimately enhancing SWB. On the one hand, based on the Group Socialization Theory (Harris, 1995), peer relationship plays a crucial role in the growth and development of adolescents, it provides opportunities for emotional expression, while peer recognition enhances their sense of self-worth. When adolescents feel accepted and respected by their peers, they are more likely to develop a positive self-image, which in turn promotes optimism (Haddow et al., 2021).

On the other hand, the present study further supports Expectancy-Value Theory, which posits that the set goal of individual behavior has an important value role for individuals (Elliot, 2006), and positive expectations for the future make individuals more sensitive to positive stimuli and enable them to experience more positive emotions (Kelberer et al., 2018). The campus peer relationship of middle school students can promote the development of their optimistic traits, and the optimistic expectations for the future can help them better cope with changes in external pressure, and develop realistic beliefs about their peer relationships through reasonable emotion regulation strategies. This, in turn, helps them adapt more successfully to campus life and improve subjective happiness and feeling (Zou et al., 2022; Krifa et al., 2022).

According to Positive Psychology, optimism is a kind of positive psychological resources, and establishing supportive interpersonal relationships can promote the development of optimism, which in turn fosters the positive growth in individual peer relationship. Middle school students with higher levels of optimism possess more flexible cognitive models and are better able to construct various positive psychological resources to cope with challenges. For example, by employing the emotion regulation strategy of cognitive reappraisal, they can reduce their perception of emotional events in the early emotional process and reduce their negative emotional experience, thus serving a “defensive” function. At the same time, cognitive reappraisal can promote their positive attitude toward life events, effectively improve the quality of life, and experience higher SWB (Gordon et al., 2016; Muazzam et al., 2021; Zhang et al., 2020).

## 5 Recommendations and limitations

This study provides both theoretical and practical insights. Theoretically, it explores the SWB of middle school students through the lens of external environment (peer relationship), personality traits and cognitive processes, thereby elucidating the underlying mechanism of SWB and enriching its theoretical framework. This study demonstrates the “peer relationship→dispositional optimism→cognitive reappraisal→SWB” pathway in middle school students, thereby extending the application of person-environment interaction theory. Practically, the findings provide guidance for educators aiming to enhance students’ SWB. Internally, educators can regulate their emotions under social pressure by fostering an optimistic disposition and personality traits among individuals, developing a positive attribution style, and adopting effective emotion regulation strategies; they can also appropriately adjust their self-perception, thereby fostering high-quality peer relationship and enhancing SWB. Externally, educators can improve students’ SWB by cultivating a supportive and harmonious school environment. These findings highlight the importance of school-based interventions focusing on peer-support enhancement, optimism training, and cognitive reappraisal skills to promote adolescents’ well-being. Clinicians and educators might integrate social skills workshops and cognitive restructuring exercises into prevention programs.

Several limitations merit consideration. First, the cross-sectional research design, which, despite being supported by previous theoretical and empirical work, precludes causal inference. Therefore, future research should employ longitudinal and experimental designs to address this limitation. Second, our methodology relied exclusively on self-reports from secondary school students, which may be subject to social desirability bias. Although the questionnaire had reverse-scored items to mitigate common methodological biases, some errors may still be present. Future research could incorporate experimental methods to obtain more accurate data. Third, this study focused solely on school-based peer relationship, dispositional optimism, and two emotion-regulation strategies, without accounting for additional environmental factors (e.g., family, community) or individual variables (e.g., cognitive ability, other personality traits). Although demographic variables (gender/age/grade) were controlled, socioeconomic status, parenting style, and academic stress may also influence these relationships and warrant examination in future studies. At last, the study sample was limited to middle school students in a single province, which may limit the universality of the findings across different cultural or regional backgrounds in China. Moreover, our findings indicate that expressive suppression did not significantly predict SWB, in contrast to Western research, thereby underscoring the cultural specificity of emotion-regulation strategies. Future research should incorporate teachers’ perspectives within the school system and exploring a broader range of underlying traits to further investigate their impact on students’ SWB.

## 6 Conclusion

This study aims to develop a chain mediation model to explore the mechanisms underlying the effect of peer relationship on SWB among middle school students. Consistent with our hypotheses, the results reveal that peer relationship exerts both direct and indirect effects on SWB. The indirect effects occur via the separate mediating roles of dispositional optimism and cognitive reappraisal, as well as through a chain mediation effect.

## Data Availability

The original contributions presented in the study are included in the article/supplementary material, further inquiries can be directed to the corresponding author/s.

## Footnotes

^1^The score of the peer relationship scale is interpreted as: the higher the score of the scale, the worse the peer relationship of the individual. Therefore, the statistical relationship between the score of the peer relationship scale and the score of other variables scale is negative. In fact, the peer relationship variables are logically positively correlated with these variables, which conforms to the expected hypothesis.
